# Ultrafast optical control using the Kerr nonlinearity in hydrogenated amorphous silicon microcylindrical resonators

**DOI:** 10.1038/srep02885

**Published:** 2013-10-07

**Authors:** N. Vukovic, N. Healy, F. H. Suhailin, P. Mehta, T. D. Day, J. V. Badding, A. C. Peacock

**Affiliations:** 1 Optoelectronics Research Centre, University of Southampton, Southampton SO17 1BJ, UK; 2 Physics Department, Faculty of Science and Technology, Universiti Malaysia Terengganu, 21300 Kuala Terengganu, Malaysia; 3 Department of Chemistry and Materials Research Institute, Pennsylvania State University, 16802 PA, USA

## Abstract

Microresonators are ideal systems for probing nonlinear phenomena at low thresholds due to their small mode volumes and high quality (*Q*) factors. As such, they have found use both for fundamental studies of light-matter interactions as well as for applications in areas ranging from telecommunications to medicine. In particular, semiconductor-based resonators with large Kerr nonlinearities have great potential for high speed, low power all-optical processing. Here we present experiments to characterize the size of the Kerr induced resonance wavelength shifting in a hydrogenated amorphous silicon resonator and demonstrate its potential for ultrafast all-optical modulation and switching. Large wavelength shifts are observed for low pump powers due to the high nonlinearity of the amorphous silicon material and the strong mode confinement in the microcylindrical resonator. The threshold energy for switching is less than a picojoule, representing a significant step towards advantageous low power silicon-based photonic technologies.

Microresonators that strongly confine light both spatially and temporally hold great potential for the construction of ultra-compact, low power, high speed photonic devices. With an appropriate choice of cavity geometry and material, these resonators can be useful for scientific and technological application in areas such as cavity quantum electrodynamics, nonlinear optics, microlasing, and biosensing^1^. Owing to the current interest in silicon photonics, a number of recent efforts have focused on crystalline silicon resonators fabricated using planar microelectronics processing technologies^2^,^3^. In particular, substantial progress has been made in the area of optical signal processing including the demonstration of all-optical logic operations^4^, modulation and switching^5^. However, to date, these devices have either made use of the thermo-optic nonlinearity^3^ or the free carrier index change^5^ to manipulate the signal so that the operational speeds are limited by the slow thermal response time, or the long free carrier lifetime. A more preferable approach would be to make use of the Kerr effect associated with the large third order nonlinearity *χ*^(3)^, which has a response time of the order of femtoseconds, thus allowing for ultrafast, broadband all-optical processing. Although Kerr nonlinear switching has been demonstrated in a range of resonator structures including GaAs planar microcavities^6^, polymer coated microspheres^7^, as well as silica microring towers^8^ and bottle microresonators^9^, it has yet to be exploited in devices fabricated from crystalline silicon materials.

The refractive index change associated with the Kerr effect depends on the intracavity intensity *I* as: *n* = *n*_0_ + *n*_2_*I*, where *n*_0_ and *n*_2_ ∝ Re[*χ*^(3)^] are the linear and nonlinear refractive indices, respectively. Although compared to silica, silicon has a large nonlinear index (~ 100 times larger), at telecommunications wavelengths near 1550 nm two-photon absorption (TPA) is significant and can result in large TPA induced free carrier index changes that dominate the operation, restricting the observation of ultrafast Kerr processes^10^. However, we have recently demonstrated the fabrication of a new class of microcylindrical resonators from our silicon fibre platform which are based on hydrogenated amorphous silicon (a-Si:H)^11^. Importantly, this material has been shown to have a nonlinear index greater than twice that of crystalline silicon, and a relatively modest TPA coefficient *β*_TPA_ ∝ Im[*χ*^(3)^], so that the overall nonlinear figure of merit (FOM) is larger^12^,^13^,^14^,^15^. Furthermore, this unique fabrication technique allows for the construction of resonators with micron sized diameters that have ultra-smooth surfaces (~ 0.1 nm RMS roughness), thus enabling small mode volumes while minimizing the scattering losses^16^.

In this paper, we present an experimental demonstration of the nonlinear Kerr effect in a silicon resonator which is fabricated from a fibre with a ~ 6 μm diameter a-Si:H core. The intensity induced increase in the refractive index manifests as a red shift of the whispering gallery mode (WGM) resonances. To deconvolve the Kerr process from the much slower thermal nonlinearity, we monitor the wavelength shifts when pumped with low duty cycle picosecond pulses as compared to a continuous wave (CW) source. The low pulsed peak powers used in the measurements indicate that the WGMs of our cylindrical resonators are well confined in a small mode volume. By exploiting the ultrafast nature of the Kerr shifting, we demonstrate all-optical modulation of the picosecond pulses with energies less than a picojoule.

## Results

### Resonator characterization

**Figure 1:**
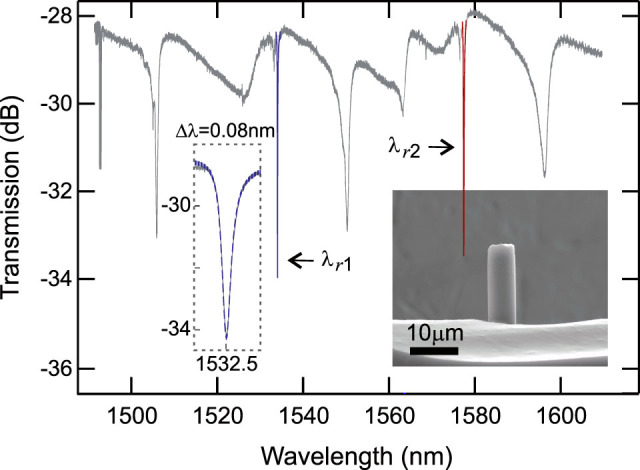
a-Si:H microcylindrical resonator transmission spectrum. Two resonances *λ_r_*_1_ ~ 1532.5 nm (blue) and *λ_r_*_2_ ~ 1577.4 nm (red) are fitted with Lorentzian line shapes (coloured curves). Insets: close up of the resonance at *λ_r_*_1_ and SEM image of the a-Si:H resonator.

The microcylindrical resonator used in our investigations was fabricated from a hydrogenated amorphous silicon optical fibre with a core diameter of 5.9 μm (see Methods). The silicon fibre platform is a particularly useful starting point as it is possible to determine the important material parameters pertaining to the resonator using standard fibre characterization techniques. Specifically, the linear loss was measured via the cutback method to be ~ 1.5 dB/cm, while the nonlinear transmission reveals a Kerr index of *n*_2_ ~ 1.7 × 10^−13^ cm^2^/W and a TPA parameter of *β*_TPA_ ~ 0.7 cm/GW, at a wavelength of 1540 nm^13^. Calculating the nonlinear figure of merit defined as^14^:
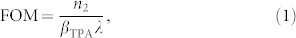
this a-Si:H fibre yields FOM ~ 1.6, which is around two to three times larger than that of crystalline silicon at this wavelength. Following the transmission measurements, the microcylindrical resonator can be revealed by simply etching the silica template away from the a-Si:H core, as shown in the inset of Fig. 1.

Figure 1 shows the transmission spectrum of the microcylindrical resonator over the wavelength range 1490 – 1610 nm (see Methods), clearly showing the sharp resonances associated with coupling to the WGMs. Two individual resonances are highlighted at the wavelengths *λ_r_*_1_ ~ 1532.5 nm and *λ_r_*_2_ ~ 1577.4 nm, in which the on resonance power drops by ~ 5 dB with respect to the off resonance transmission. The loaded quality factors *Q_l_* = *λ_r_*/Δ*λ*_FWHM_ are determined by fitting the resonances with Lorentzian line shapes (blue and red curves). From this analysis we determine the full width at half maximum (FWHM) bandwidths of the two resonances as Δ*λ*_FWHM1_ = 0.08 nm and Δ*λ*_FWHM2_ = 0.13 nm, which yields *Q_l_*_1_ = 1.9 × 10^4^ and *Q_l_*_2_ = 1.2 × 10^4^, respectively.

### Resonance wavelength shifting via Kerr nonlinearity

**Figure 2:**
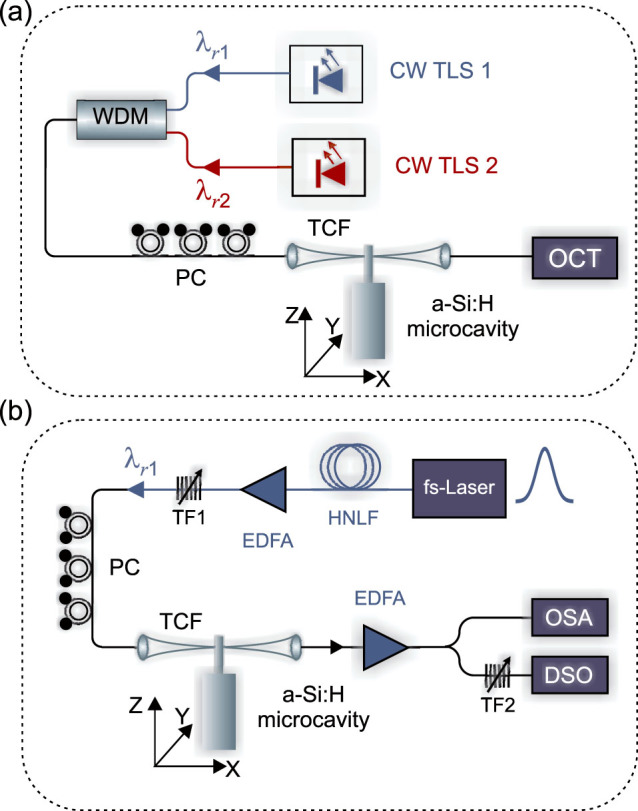
Experimental set-ups to measure nonlinear resonance wavelength shifting. (a) CW pump-probe set-up. CW TLS: CW tunable laser sources. WDM: wavelength-division multiplexer. PC: polarization controller. TCF: tapered coupling fibre. OCT: optical component tester. (b) Pulsed pump set-up. HNLF: highly nonlinear fibre. TF: tunable filters. EDFA: Erbium doped fibre amplifier. OSA: optical spectrum analyzer. DSO: digital sampling oscilloscope.

**Figure 3:**
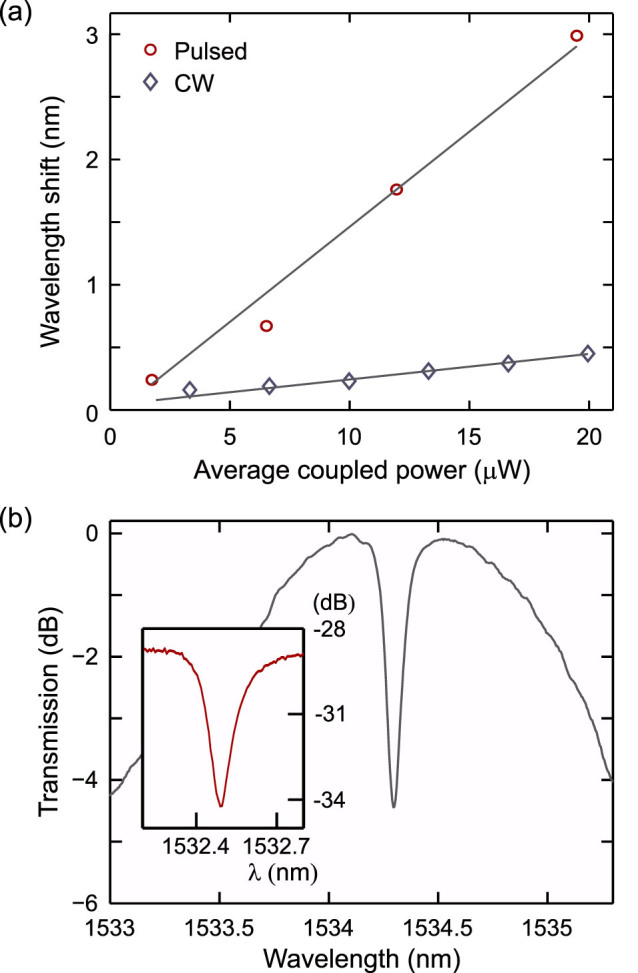
Measurements of nonlinear resonance wavelength shifting. (a) Wavelength shift when pumping the resonance at *λ_r_*_1_ as a function of average coupled power for CW (diamonds) and pulsed (circle) pumps. (b) Pulse spectrum showing coupling to the resonator at *λ_r_*_1_ at an average power of 12 μW, corresponding to a wavelength shift of Δ*λ* = 1.76 nm from the cold cavity position (as shown in the inset).

To investigate the wavelength shift of the resonances associated with the nonlinear effects we employed two different experimental set-ups as shown in Fig. 2. These two set-ups allowed us to compare the wavelength shifting resulting from (a) a CW pump and (b) a high peak power, low duty cycle picosecond pulse source. In the first experiment of Fig. 2(a), we positioned a tunable CW pump (CW TLS 1) on *λ_r_*_1_, then used a second CW probe (CW TLS 2) at low power to scan over the resonance at *λ_r_*_2_. Both the pump and probe beams were coupled into the a-Si:H resonator via a single tapered silica fibre and the output spectrum was monitored using an optical component tester (OCT) (see Methods). The resonant wavelength shift as a function of CW power coupled into *λ_r_*_1_ is plotted in Fig. 3(a) (diamonds). For the second experiment of Fig. 2(b), the first stage involved generating a pulse source positioned on *λ_r_*_1_. This was achieved by pumping a highly nonlinear fibre with a femtosecond fibre laser (720 fs, 40 MHz), which produced a broadband supercontinuum that could be filtered (TF1) to yield 2.2 ps (FWHM) pulses at the desired wavelength. As the spectral bandwidth of the pump pulses is broader than the cavity resonance, the wavelength shift of the resonance can be monitored via the position of the absorption dip on the output pulse spectrum, as illustrated in Fig. 3(b) (inset shows the cold cavity position for comparison). However, to ensure that the measured shift was occurring due to the presence of the pump, we also analyzed the output in the time domain using a second, narrow-band (0.1 nm), tunable filter (TF2) coupled with a high speed 30 GHz photodetector and digital sampling oscilloscope (DSO) (see Methods). By monitoring the pulses on the DSO as TF2 was tuned, the wavelength shift could be established from the point where the maximum reduction in the pulse amplitude was observed (i.e., when most of the light is coupled into the resonator). The corresponding wavelength shift as a function of coupled average power is also plotted in Fig. 3(a) (circles), together with the CW data.

The wavelength shifts in Fig. 3 both show a linear increase as a function of average coupled power, as expected for the thermal and Kerr nonlinear processes^7^. For the pulsed pump, the results yield a resonance wavelength shift of 3 nm at an average coupled power of ~ 20 μW. Accounting for the spectral filtering due to the narrow resonator bandwidth, which broadens the pulse widths to ~ 25 ps, this corresponds to an estimated peak power coupled into the resonator of only ~ 20 mW. In comparison, for the CW pump only a small 0.45 nm resonance shift was observed for the maximum coupled power. This small shift can be entirely attributed to the thermal nonlinearity resulting from the material absorption, and the moderate size reflects the relatively low losses of the a-Si:H material^17^. It is generally considered that when pumping with a pulsed source with a period that is shorter than the thermal response time, the temperature eventually stabilizes at a value which only depends on the average power^4^. However, before we attribute the order of magnitude difference in the two resonance shifts to the Kerr nonlinearity we investigate the relative contributions to the wavelength shifting in more detail.

The refractive index change that occurs in a silicon resonator depends on three processes: (i) the thermo-optic effect, (ii) the Kerr nonlinearity, and (iii) the free carrier refraction. Considering each of these contributions, the wavelength shift associated with the circulating power *P_c_* can be defined as^18^:
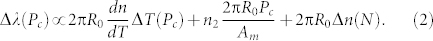
Here *R*_0_ is the cylinder radius, *A_m_* is the mode area, and *P_c_* is proportional to *Q_l_* as: *P_c_* = *Q_l_λP_in_*/(4*π*^2^*R*_0_*n*_eff_), where *n*_eff_ is the effective refractive index associated with the WGM and *P_in_* is the coupled input peak power. In the first term relating to the thermal nonlinearity, *dn*/*dT* = 2.3 × 10^−4^ K^−1^ is the thermo-optic coefficient of a-Si:H^19^ and Δ*T*(*P_c_*) is the temperature change due to the fraction of *P_c_* that is absorbed, both through linear and nonlinear (TPA and free carrier absorption (FCA)) processes (see Methods). In the final term related to the free carrier refraction, Δ*n*(*N*) is the refractive index shift induced by the free carrier density *N*(*P_c_*), given by Δ*n*(*N*) = [−8.8 × 10^−22^*N* − 8.5 × 10^−18^*N*^0.8^](*λ*/1.55)^2^, where *N* is determined by the usual rate equation^13^ and expressed in units of cm^−3^, while *λ* is in μm. However, as the negative sign in Δ*n* induces a blue shift, owing to the large observed red shift we expect this term to be much smaller than the Kerr shift. It is important to note that *A_m_*, which determines the WGM mode volume *V_m_* ≈ 2*πR*_0_*A_m_*, and thus appears in all three terms, depends on the resonator mode height. Although the fibre geometry does not offer any structural confinement of the mode in the vertical dimension *z*, it has been shown that cylindrical resonators can support modes that are highly localized, as evidenced by the relatively high measured *Q* factors^20^. Using the analysis of Ref.^20^ we have previously estimated the mode height to be ~ 5 μm, from which the mode area can be calculated by solving the Helmholtz equation in cylindrical coordinates^11^. However, as the linear and nonlinear material parameters of the resonator have already been determined through the transmission measurements of the a-Si:H core fibre, we can use the best fit for the wavelength shifts to obtain a more informed estimate of the height.

In order to fit the wavelength shifts, we start by considering the thermal dynamics due to the use of a pulse train. By calculating the power absorbed for the incoming pulses *P*_abs_(*t*), we can determine the evolution of the temperature rise (see Methods). As in Ref.^21^, we find that as the 25 ns pulse spacing is shorter than the thermal response time *τ*_th_ ~ 7.5 μs of the a-Si:H resonator^17^, initially the temperature rises in a step like fashion until it reaches a steady state for times greater than 40 μs. Thus we can assume that for a given average power, the thermal wavelength shift is fixed and use Eq. (2) to fit the total shift when the pulse is on. We find that the best fit for the pulsed pump wavelength shifts occurs for *V_m_* ~ 15 μm^3^. This small mode volume implies that the modes in our cylindrical resonators are indeed well confined in *z* to a height of ~ 1.5 μm. Although this height is slightly smaller than the initial prediction, this is not unexpected owing to the high material and coupling losses of our a-Si:H resonator compared to the system described in Ref.^20^. In addition to the volume, the modelling also reveals that for the maximum average power of 20 μW the estimated steady state thermal shift including the effects of nonlinear absorption is Δ*λ*_th_ ~ 0.6 nm, and the free carrier refraction induced shift is negligible. Thus we can conclude that the majority of the large 3 nm wavelength shift associated with the pulsed pump is indeed due to the large Kerr nonlinearity of the resonator, which is in accordance with the high FOM of our a-Si:H material when compared to crystalline silicon^10^. Importantly, as for a given input power the nonlinear resonance shift is proportional to^9^ *n*_2_*Q*^2^/*V_m_*, the combination of a large *n*_2_ and small *V_m_* makes these resonators ideal for ultra-low power signal processing. In future work we will look to introduce a degree of structural confinement in the axial direction by shaping the a-Si:H cylinders into a bottle geometry, as often employed for the fabrication of resonators based on standard silica fibres^9^. Such three-dimensional confinement should result in reduced leakage losses, and hence increased *Q* factors, as well as allow for some tuning of the resonator parameters such as the mode volume^22^.

### Kerr-induced optical control

**Figure 4:**
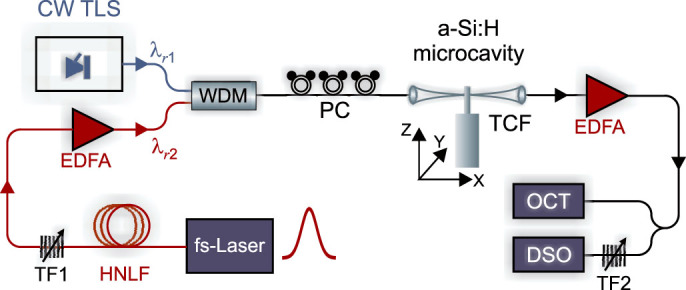
Set-up for all-optical modulation using the Kerr effect. Labels as defined for Fig. 2.

**Figure 5:**
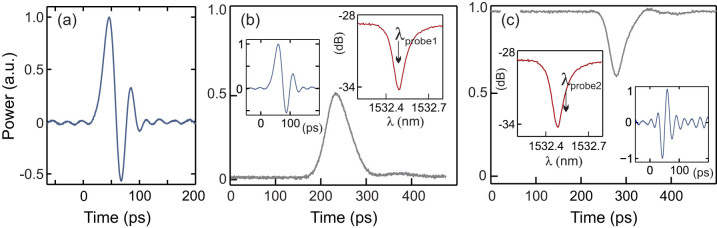
Ultrafast Kerr all-optical modulation. (a) Impulse response of 2.2 ps pump. (b) Filtered modulated bright pulse. Insets: blue curve is the temporal impulse response, red curve shows the probe position with respect to the cold cavity resonance dip. (c) Same as for (b) but for modulated dark pulse.

To exploit the large Kerr wavelength shift and demonstrate its use for ultrafast all-optical processing, we modified the set-up in Fig. 2(b) to include a low power CW probe for modulation via the train of pump pulses. Fig. 4 shows the experimental configuration which, aside from the evanescently coupled silicon resonator, is entirely fiberized so that it is quite robust with the potential to be extremely compact. Similar to the set-up in Fig. 2(a), the two sources were tuned to the resonances *λ_r_*_1_ (CW probe) and *λ_r_*_2_ (pulsed pump) and launched into the tapered fibre. At the output, the tunable filter (TF2) is now used to isolate the probe which is again monitored via the DSO. Unfortunately, the picosecond durations of the pulses cannot be accurately resolved by this bandwidth limited detection system, as illustrated by the impulse response of the 2.2 ps pump pulse shown in Fig. 5(a). Thus to monitor the modulated outputs a RF Bessel Thomson (fourth order low-pass) filter was applied to remove the high frequency components (3 dB cut-off at 7.465 GHz), which results in the pulses appearing longer than in reality. The filtered probe signals at two different input wavelengths are shown in Figs. 5(b) and (c), demonstrating the capability to achieve both bright and dark modulated data streams, with the corresponding impulse responses displayed in the insets (blue curves) for completeness. The picosecond on-off switching times observed in Fig. 5 provide final confirmation of the ultrafast nature of the resonance shifting due to the pulsed pump. As the Kerr response time is subpicosecond, in these experiments we expect the switching time to be determined by the bandwidth of the cavity at the pump resonance^4^ *λ_r_*_2_, so that the modulated pulse durations should be around ~ 25 ps. For the bright pulse in Fig. 5(b), the probe was launched into the tapered fibre on resonance (*λ*_probe1_ = 1532.5 nm, as illustrated by the position of the arrow on the red curve in the inset) so that the presence of the pump pulse in *λ_r_*_2_ caused the probe to be shifted out of the resonance, i.e., switching the light on. The modulation extinction ratio determined as the difference between the on-off transmitted optical probe power was 3 dB, though this could be improved by coupling to WGMs with larger extinctions as demonstrated in Ref.^11^. In contrast, the dark pulse in Fig. 5(c) corresponds to when the probe is launched slightly off resonance *λ*_probe2_ = 1532.6 nm so that the presence of the pump shifts the resonance towards the probe, switching it off. In this instance the extinction is slightly reduced to 2 dB, which is largely due to the difficulty in positioning the probe so that it is switched into the resonance minimum. We note that the impulse response for the dark pulse appears slightly shorter due to the shorter fall time (7 ps, versus a rise time of 18 ps) of the detector. Significantly, the optical modulation displayed in Figs. 5(b) and (c) was realized for an average coupled pump power of only ~ 11 μW, corresponding to ~ 10 mW of peak power. However, the switching threshold was as low as ~ 5 μW, which is an order of magnitude lower than the power used to demonstrate Kerr switching in a silica bottle resonator^9^. In comparison to the crystalline silicon-based ring resonators devices based on free carrier refraction, the estimated pump energy for this demonstration of only 0.3 pJ represents a reduction of almost two orders of magnitude in switching energy^5^.

## Discussion

The observation and characterization of the nonlinear Kerr resonance wavelength shifting shown in Fig. 3 has been enabled by two key properties of our resonator: (i) the high nonlinear index of the a-Si:H material and (ii) the small mode volume of the few-micron diameter resonator, both signature features of our unique fabrication method which starts from the silicon fibre platform. The ability to exploit the subpicosecond response times of the Kerr nonlinearity is an important step towards developing truly high speed all-optical processing devices using silicon technologies. Importantly, growing interest in the CMOS compatible highly nonlinear a-Si:H material for wafer based technologies has led to some similar observations of Kerr-induced nonlinearities in ring-resonators^23^,^24^, and we anticipate that with continued advancements in the deposition and fabrication of a-Si:H devices research in this area will flourish.

By exploiting the large wavelength shifts associated with the high nonlinear index, we have demonstrated all-optical control with a picosecond scale on-off switching time governed by the resonator bandwidth. As well as modulating the signal as we have shown here, true switching capabilities could be realised by using a second tapered fibre as a “bus” or “drop port” to route the signal via the presence or absence of the pump^9^. Notably this amplitude modulation represents an order of magnitude increase in the device speed compared to previously reported demonstrations in crystalline silicon resonators that have utilized free carrier refraction^5^, and that the switching threshold has been achieved with a power level of only a few microwatts, which corresponds to subpicojoule pulse energies. Moreover, these modulation speeds could easily be reduced further by simply decreasing the quality factor *Q*, though this would come at the expense of an increased energy threshold. As well as providing a route towards compact, low power, high speed all-optical processing devices, we expect these novel resonators to also allow for more fundamental investigations of low threshold nonlinear light-matter interactions.

## Methods

### Silicon resonator fabrication

The silicon core fibre was fabricated by depositing a-Si:H inside a 5.9 μm diameter silica capillary using a high pressure microfluidic chemical technique. In this process, a mixture of silane and hydrogen (SiH_4_/H) are forced to flow through the capillary under high pressures ~ 35 MPa, while being heated in a tube furnace to temperatures ~ 400 °C. At this temperature the dissociation of hydrogen from the precursor is not complete so that some remains in the core, which is confirmed by the presence of the S–H stretching (S) mode in micro-Raman measurements conducted at 633 nm using a Renishaw inVia Raman spectrometer. The incorporation of hydrogen in the amorphous material is important as it acts to passivate the dangling bonds, greatly reducing the optical transmission loss. Following the fibre fabrication, the microcylindrical resonator is formed by etching the silica cladding away from the a-Si:H core using a buffered hydrofluoric acid (HF) solution (volume ratio 20:1 of 40% ammonium fluoride in water and 49% HF in water), before rinsing with deionized water.

### Resonator characterization

A single tunable external cavity CW laser source (Tunics Plus) with a linewidth of ~ 400 kHz was coupled to the a-Si:H resonator using a tapered single mode silica fibre with a waist diameter of 1 – 2 μm (see Fig. 2(a)). Polarization control was placed before the taper to selectively couple into the transverse electric (TE) modes of the cylindrical resonator. The spectral output from the taper was monitored using an optical component tester (Yenista CT400) with a 1 pm resolution. For the high speed pulse measurements, a femtosecond fibre laser (onefive) with a 720 fs (FWHM) duration and a 40 MHz repetition rate was used as the starting source. The modulated output was monitored using a digital sampling oscilloscope (Agilent 83480A) via a 30 GHz photodetector (Agilent 83485B).

### Modelling of the thermal dynamics

To numerically simulate the thermal dynamics we apply the formulism outlined in Ref.^25^. The absorbed power *P*_abs_ can be calculated as: *P*_abs_(*t*) = [*γ_l_* + *γ*_TPA_ + *γ*_FCA_]*U*(*t*), where *γ_l_*, *γ*_TPA_, and *γ*_FCA_ are the loss rates for the linear and nonlinear (TPA and FCA) processes, respectively, and *U*(*t*) is the energy stored in the resonator. The loss rates are evaluated using the material parameters established from the transmission measurements of the a-Si:H core fibre. The time-dependent temperature rise *T*(*t*) associated with *P*_abs_(*t*) is then obtained by solving the differential equation^26^:
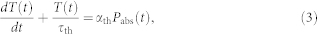
where *α*_th_ is the thermal absorption rate. The integration of Eq. (3) is conducted using an ordinary differential equation solver based on a Runge-Kutta method.

## Author Contributions

N.V., N.H., and A.C.P. designed the research. N.V., F.S., P.M., and N.H. carried out the experiments, N.V. and A.C.P. conducted the analysis. The silicon fibre was fabricated by T.D.D. under the supervision of J.V.B. The manuscript was written by A.C.P. and N.V.; all authors contributed to revision of the manuscript.
